# Fabrication and Characterization of a Biomaterial Based on Extracellular-Vesicle Functionalized Graphene Oxide

**DOI:** 10.3389/fbioe.2021.686510

**Published:** 2021-06-09

**Authors:** Julia Driscoll, Anuradha Moirangthem, Irene K. Yan, Tushar Patel

**Affiliations:** Department of Transplantation, Mayo Clinic, Jacksonville, FL, United States

**Keywords:** extracellular vesicles, graphene, therapeutics, biomaterials, toxicology

## Abstract

Mesenchymal stem cell (MSC) derived extracellular vesicles (EV) are emerging as acellular therapeutics for solid organ injury and as carriers for drug delivery. Graphene-based materials are novel two-dimensional crystal structure-based materials with unique characteristics of stiffness, strength and elasticity that are being explored for various structural and biological applications. We fabricated a biomaterial that would capture desirable properties of both graphene and stem cell derived EV. Metabolically engineered EV that express azide groups were cross-linked with alkyne-functionalized graphene oxide (GO) via a copper catalyzed alkyne-azide cycloaddition (CuAAC) reaction. The crosslinking between EV and GO was accomplished without the need for ligand expression on the metal. Scanning electron and fluorescence microscopy demonstrated excellent cross-linking between EV and GO. Biological effects were assessed by phagocytosis studies and cell viability studies. The uptake of GO or sonicated GO (sGO) resulted in a durable pro-inflammatory immune response. Cell studies further showed that crosslinked GO-EV scaffolds exhibited cell-type dependent cytotoxicity on liver cancer cells whereas there was minimal impact on healthy hepatocyte proliferation. *In vitro*, neither GO-EV nor sGO-EV induced DNA strand breaks. *In vivo* studies in zebrafish revealed gross developmental malformations but treatment-induced mortality was only seen with the highest doses of GO-EV and sGO-EV. With these advantages, this engineered biomaterial combining the versatility of graphene with the therapeutic effects of MSC-EV has potential for applications in tissue engineering and regenerative medicine.

## Introduction

Extracellular vesicles (EV) are membrane-bound nanovesicles that can be released from different cell types and are ubiquitous in biological fluids. EV can play an essential role in intercellular communication through the unilateral transfer of their cargo to recipient cells (Yáñez-Mó et al., 2015). Delivery of their cargo to recipient cells is facilitated by membrane proteins that promote cellular uptake by recipient cells. These properties facilitate the use of EVs as delivery agents for therapeutics. Moreover, EV derived from mesenchymal stem cells (MSC-EV) have potential as an acellular therapeutic, due to their intrinsic beneficial therapeutic properties such as promoting tissue repair (Reis et al., 2012; Yang et al., 2015; Haga et al., 2017). The cargo of EV can be modified to enhance their capability and utility as acellular therapeutics. Modification of the EV cargo can be accomplished by either loading the EV with therapeutic molecule(s) of interest or through protein engineering manipulations of the parent cells (Tian et al., 2014; Li et al., 2018; Matsuda et al., 2019).

Graphene is a carbon-based material that has garnered much recent attention in the scientific community. This unique material is comprised of carbon atoms arranged in a two-dimensional honeycomb lattice. The delocalization of one of the electrons in each carbon atom endows graphene with a high tensile strength and exceptional thermal and electrical conductivity that make it attractive for use in a variety of biomedical applications (Geim, 2009). Furthermore, the development of graphene-based drug and nucleic acid delivery vehicles is supported by the high surface area of graphene that allows for the loading of pharmaceutical agents or biological macromolecules. The surface chemistry of graphene can be modified to generate derivatives with different physicochemical properties. Oxidation of graphene generates graphene oxide (GO), a hydrophilic biomaterial, whereas reduction of GO results in the formation of reduced GO. The surface of graphene and its derivatives can be altered by covalent as well as non-covalent modifications. The most common surface modifications include the addition of functional group(s) to enable cycloaddition reactions, the conjugation of polymers to increase the biocompatibility of the biomaterial and the attachment of antibodies to enable the targeted delivery of the biomaterial (Mei et al., 2015; Xu et al., 2016; Zheng et al., 2016). The versatility of graphene enables its use for a variety of different biomedical applications. Indeed, graphene and its derivatives have already been effectively used for biosensing, drug and nucleic acid delivery, photothermal and photodynamic therapy for cancer treatment, and tissue engineering (Robinson et al., 2011; Hu et al., 2012; Lee et al., 2016; Shin et al., 2016; Vinothini et al., 2019).

The overall goals of our study were to develop a composite graphene-based biological material that would allow us to further exploit the use of EV for drug delivery or tissue repair. We postulated that the functional applications of EV-based therapeutic applications could be extended by combining them with the versatile properties offered by the application of graphene-based materials. Using a protein engineering and biorthogonal click conjugation strategy, we generated a biological graphene nanoparticle by conjugating EV to graphene oxide. This new biomaterial (GO-EV) can be readily generated, retains biological effects of EV, and could support the development of new applications in tissue engineering, repair and regenerative medicine.

## Materials and Methods

### Cells and Cell Culture

Human bone marrow derived-mesenchymal stem cells (hBM-MSC) were purchased from Lonza (Walkersville, MD) and maintained in MSC basal media supplemented with L-glutamine, gentamicin sulfate, amphotericin and mesenchymal cell growth supplement (Lonza; Walkersville, MD). After the third passage, the cells were cultured in vesicle-depleted media. Vesicle depletion from media was performed by tangential flow filtration (TFF) using a sterile 500 kDa molecular weight cut off MidiKros filter lined with a modified polyethersulfone membrane (Repligen; Waltham, MA). The permeate, containing the vesicle-depleted MSC media, was collected and passed through a 0.22 μm filter before storing at 4°C. Human hepatocytes (HH; Sciencell, United Kingdom) and PLC cells (ATCC; Manassas, VA) were cultured in untreated plates. KMBC (provided by Dr. Gregory Gores, Mayo Clinic), HepG2 and Hep3B (ATCC; Manassas, VA) cells were cultured in collagen-coated plates. The aforementioned cells were maintained in Dulbecco’s modified eagle media (DMEM) high glucose media supplemented with 1% penicillin-streptomycin and 10% fetal bovine serum (FBS). HL-60 promyeloblasts (ATCC; Manassas, VA) were cultured with Iscove’s modified Dulbecco’s medium supplemented with 20% FBS in T75 flasks. RAW264.7 murine macrophages (ATCC; Manassas, VA) were cultured with DMEM high glucose media supplemented with 10% FBS.

### Isolation of EV

MSC-conditioned media (MSC-CM) was collected from azide-tagged or untagged MSC in culture and centrifuged to remove debris and apoptotic bodies. The MSC-CM was first centrifuged at 300 × g for 5 min at 4°C; the supernatant was transferred to new tube and centrifuged at 2,000 × g for an additional 30 min. The supernatant was transferred to a 250 mL reservoir for isolation by TFF with a 500 kDa molecular weight cut-off filter. The flow rate was maintained at 53 mL/min with a sheer rate that did not exceed 3,000 for the duration of the isolation process. The MSC-CM was concentrated 5 times to reduce the volume to 5 mL, diafiltrated 5 times with PBS and further concentrated to attain a final volume of approximately 2–5 mL. The filter was washed once with PBS prior to loading with subsequent batches of MSC-CM. Fifty microliters of untreated EV or azide-tagged EV (az-EV) were diluted in PBS (1:100) for quantitation of particle size and concentration using the nanosight (Malvern Panalytical, United Kingdom). A BCA assay (Thermo Fisher Scientific, Waltham, MA) was used to quantitate proteins, with concentrations of EV extrapolated from a standard curve that was constructed using a 4-parameter fit. Aliquots of the isolated EV were stored at 4°C for later use.

### Generation of GO-EV

MSC were treated with 50 μM of N-azidoacetylmannosamine-tetraacetylated (Ac_4_ManNAz; Kerafast, Boston, MA) for 72 h. Az-EV were isolated from Ac_4_ManNAz-treated MSC. Alkyne functionalized graphene oxide (GO) was obtained from Nanocs (cat no. GO1-AK-1, New York, NY), and characteristics are reported in Supplementary Table 1. GO or sonicated GO (sGO) was covalently bound to az-EV by copper-catalyzed click chemistry. sGO was generated as described by Campbell et al. (2019) Briefly, GO was subjected to ultra-low power sonication for 20 min (Branson Sonifer 150; Danbury, CT), followed by centrifugation for 5 min at 2,000 × g to remove large aggregates, and repeat sonication of the supernatant for 30 min. GO-EV or sGO-EV were generated by incubating equivalent amounts of EV and GO or sGO at room temperature (RT) in the dark for 30 min using the reaction buffer kit (Click Chemistry Tools, Scottsdale, AZ). The reaction products were centrifuged at 14,000 × g for 10 min. The supernatants containing the reaction buffers and unbound substrates were removed by centrifugation and the GO-EV and sGO-EV pellets were resuspended in PBS and stored at 4°C for later use.

### Fluorescence Imaging

Untreated EV and az-EV were diluted with PBS to a concentration of 2 × 10^10^ particles/mL and stained with 10 μM/L DiI (Life Technologies; Carlsbad, CA) for 60 min with periodic mixing, followed by ultracentrifugation at 100,000 × g for 70 min at 4°C. The supernatants were removed, and the pellets were resuspended in a working volume of PBS. Click chemistry reactions were performed using 5.7 μg GO and 2.9 μg DiI stained unmodified or az-EV. DiI stained unmodified EV-GO or az-EV-GO were transferred to microscope slides and visualized using fluorescence microscopy (Life technologies; Carlsbad, CA).

### Spectral Analysis

GO or sGO were diluted in PBS to desired concentrations (200–800 μg/mL). The samples were transferred to quartz microcuvettes and absorbance spectrophotometry was performed using a Beckman Spectrophotometer (Beckman Coulter; DU800; Brea, CA). PBS was used as a blank.

### Scanning Electron Microscopy (SEM)

GO-EV or sGO-EV were generated using 200 μg/mL azide tagged EV and 200 μg/mL GO or sGO by click chemistry. The reaction products underwent centrifugation at 14,000 × g for 10 min, the supernatants were discarded and the pellets were resuspended in a 4% glutaraldehyde/0.1 M PBS fixative. Small volumes of 200 μg/mL GO and sGO were aliquoted and centrifuged at 14,000 × g for 10 min. The supernatants were removed and the pellets were resuspended in water. The washing step was repeated once more to remove any residual salts from the samples. After the final wash step, the GO and sGO samples were air-dried on poly-L lysine coated coverslips after which they were mounted on an aluminum stub and sputter coated (E5100 SEM Sputter Coater, Bio-Rad, Hercules, CA) for 1 min with gold-palladium.

After the final wash, the GO-EV and sGO-EV samples were centrifuged at 14,000 × g for 10 min and fixed for 1 h at 4°C in Trump’s fixative (4% formaldehyde, 1% glutaraldehyde in 1.0 M phosphate buffer with a pH of 7.2). Once fixed, the samples were transferred to poly-L lysine coated coverslips. First, they were washed with PBS, followed by water and subsequently dehydrated twice through a graded series of ethanol concentrations (10, 30, 50, 70, 90, 95, and 100%). The samples were then subjected to critical point drying (CPD) with liquid CO_2_ (EMS 3100 CPD; Electron Microscopy Sciences; Hatfield, PA). The samples were then sputter coated using the method described above. SEM was performed by the Mayo EM core facility using a S-4700 cold field emission SEM set to a 5 kV accelerating voltage (Hitachi; Tokyo, Japan).

### Cytotoxicity Assays

Cells were seeded in a 96 well plate and allowed to attach overnight. The following day, the media was aspirated and the cells were washed once with PBS. The PBS was removed and 100 μL of vesicle-depleted DMEM high glucose media was added to each well. The cells were then treated with the following: PBS (control), 0.4 μg of GO, sonicated GO (sGO), GO-EV or az-EV. To account for the background absorbance due to graphene, additional wells without cells were treated with similar amounts of GO or GO-EV. Cell viability was assessed using an MTS assay (Promega; Madison, WI) at 24–96 h post-treatment. Briefly, 20 μL of MTS reagent was added to each of the wells and the plates were maintained in complete darkness for 2 h at 37°C. The absorbance was measured at 490 nm to assess cell viability. Each treatment condition consisted of 4 technical replicates. The percent viability was calculated for each treatment condition and normalized to the control group.

### DNA Damage Assay

An Oxiselect Comet assay (Fisher; Hampton, NH) was performed to assess the genotoxicity in HL-60 cells. Cells (100,000/well) were seeded in a 24 well plate, then incubated for 24 h with PBS (diluent control), 40 μg/mL GO-EV or sGO-EV or for 1 h with 20 μm etoposide (positive control). Cells were centrifuged at 600 × *g* for 2 min, and the pellets resuspended in ice cold PBS. Alkaline electrophoresis was performed and cells were visualized using fluorescence microscopy (EVOS FL; Invitrogen, Carlsbad, CA). Each treatment was performed in triplicate. Tail length quantitation and analysis was performed using the OpenComet software (CometBio, Chicago, IL). At least 15 images from each replicate were captured. The tail length quantitation was performed using the OpenComet software (CometBio, Chicago, IL).

### Assessment of Developmental Toxicity

Zebrafish (*Danio rerio*) were obtained from the Zebrafish International Resource Center (Eugene, OR) and housed in the Mayo Clinic Jacksonville zebrafish facility. The zebrafish were fed live brine shrimp (*Artemia nauplii*) twice daily and also received dry flakes (pellets) once a day. A single male and female zebrafish were placed on opposite sides of a spawning aquarium, equipped with a separator and a mesh bottom to capture the embryos. The following morning, the separator was removed and the embryos were collected after 30–60 min and subsequently rinsed with embryo water (EW) (5 nM NaCl, 0.17 nM KCl, 0.33 nM CaCl^2^, 0.33 MgSO^4^, 0.00001% methylene blue). The fertilized embryos were transferred to single wells of a 96 well plate and maintained in 100 μL of EW. At 24 h post-fertilization (hpf), the EW was replaced with EW containing phenylthiourea 10% v/v PBS, 10 μg GO, 1 μg or 10 μg sGO, 1 or 10 μg GO-EV, 1 μg or 10 μg sGO-EV, 1 μg or 10 μg az-EV or nothing else. The treatments were refreshed every 24 h. Dechorionation was monitored every 3 h from 45 to 85 hpf. The heart rates of the zebrafish were recorded at 48, 72, 144, and 168 hpf. Furthermore, the zebrafish were examined for malformations and their survival was noted.

### Visualization of EV-GO Uptake

One milliliter of az-EV was stained with an equal volume of 4x PKH67 dye (Sigma, St. Louis, MO) for 30 min with periodic mixing, after which the labeling reaction was terminated by the addition of 1 mL of 1% BSA. The az-EV underwent ultracentrifugation at 100,000 × g for 70 min at 4°C, after which the supernatant was removed and the pellet was resuspended in PBS. Click chemistry reactions were performed with PKH67 labeled EV and GO or sGO. RAW264.7 cells (10,000/well) were seeded onto a FluoroDish (Fisher; Hampton, NH) and allowed to attach overnight. The media was aspirated and the cells were washed once with PBS. One and a half milliliters of phenol red-free media with 50 μL of PKH67 stained GO-EV or sGO-EV was added on top of the cells. Bright field and fluorescence channel images were captured every 5 min using the Nanolive 3D cell explorer (Nanolive, Switzerland). Imaging was terminated once the cell(s) had successfully internalized the biomaterials.

### Cytokine Assays

RAW264.7 cells (20,000/well) were seeded on a 96 well plate and allowed to attach overnight. The following day the cells were treated with PBS or 0.4 μg GO-EV for 3 h. The media was collected and centrifuged at 1,500 rpm for 10 min. The supernatant was collected and utilized to perform a mouse 31-plex cytokine and chemokine panel (Eve technologies, Alberta, Canada). For TNF-α assays, cells were treated with PBS, 0.4 μg GO, sGO, GO-EV, sGO-EV or az-EV for 24 h. Samples were diluted in PBS. TNF-α assays were performed by a high sensitivity TNF-α ELISA (Thermo Fisher Scientific, Waltham, MA) according to the manufacturer’s protocol, using a FLUOstar Omega plate reader (BMG Labtech, Germany) to measure absorbance. A four-parameter fit standard curve was generated using RStudio. For cytokine assays, four technical replicates were included for each treatment condition.

### Statistical Analysis

Data are reported as the average ± the standard deviation from studies performed using an appropriate number of replicates, or as otherwise indicated. For the cytokine and chemokine assays, the fold change in average concentration between treated and control cells was calculated. Comparisons across groups was performed by Student’s *t*-test.

## Results

### Generation of Bioengineered EV

**FIGURE 1:**
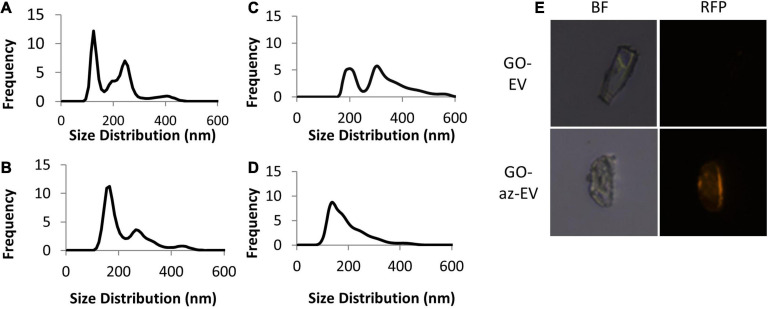
Characterization of nanoparticles. The size distribution of **(A)** unmodified MSC-EV, **(B)** azide tagged MSC-EV (az-EV), **(C)** graphene oxide (GO) and **(D)** sonicated GO (sGO). **(E)** Bright field and fluorescence microscopy images of DiI stained plain (EV) and azide tagged (az-EV) MSC-derived EV covalently bound to graphene oxide (GO) were captured with a 40x magnification.

Cell culture media was harvested from the media of early passage (passage 4–5) MSC-EV were isolated using TFF. A cell glycoprotein engineering approach involving Ac_4_ManNAz treatment was used to metabolically modify MSC. These cells integrate azide-bearing biomolecules such as amino acids and saccharides into the multivesicular bodies and can thereby introduce active azides as reaction sites on EV released by these cells. The metabolic engineering of these EVs can be performed without exposure of the cell to toxic agents, whilst maintaining their biochemical integrity and viability. Ac_4_ManNAz treated cells released EVs with azide tags. These engineered az-EV have a slightly greater mean diameter compared with unmodified EV, although the overall size distribution profile was very similar (Figure 1). A larger number of EVs and protein content (Table 1) were obtained from engineered cells compared with untreated controls.

**TABLE 1:** Size and protein characterization of unmodified and azide tagged MSC-EV.

	**[Particles] (p/mL)**	**Average particle diameter (nm)**	**Protein concentration (μg/mL)**
EV	8.03 × 10^8^	221.8	1189.7
az-EV	9.58 × 10^10^	224.3	1590.0

### Fabrication of GO-EV

Using bio-orthogonal click conjugation, az-EV were cross-linked to alkyne-functionalized GO. The reaction was catalyzed using copper through CuAAC cycloaddition. To determine if the EV were capable of binding to GO, fluorescently stained EV were reacted with GO. Fluorescence microscopy revealed DiI stained az-EV bound to GO, indicating successful conjugation. Furthermore, EV that lacked the azide tag were unable to bind to GO. Studies using alkyne-functionalized GO revealed size variations and large GO particles. To achieve size homogenization, GO was further sonicated prior to conjugation with EV for some studies. The sonicated GO (sGO) particles exhibited reduced diameters and a more homogenous size distribution profile in comparison to plain GO particles (Figure 1).

### Characterization of GO-EV Based Biomaterials

**FIGURE 2:**
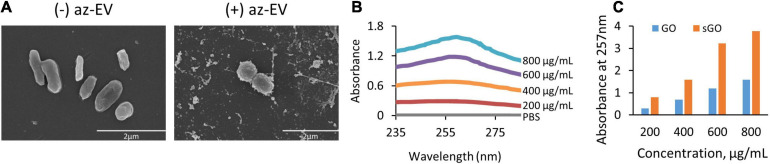
Visualization and absorbance scanning of GO biomaterials. **(A)** Scanning electron microscopy images of GO and GO-EV were captured at 25 and 20 k magnification, respectively. **(B)** Absorbance scanning of GO dilutions over 200–300 nm revealed peak absorbance at 257 nm. **(C)** Absorbance of GO and sGO dilutions at 257 nm.

**FIGURE 3:**
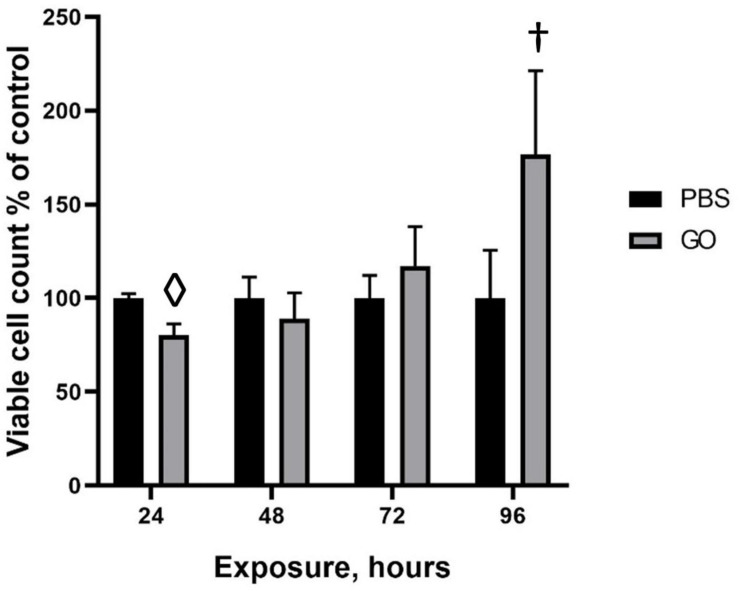
Viability of HepG2 cells in response to GO exposure. To assess the cytotoxic effects of transient exposure of cells to GO-based biomaterials, HepG2 cells were treated with 0.4 μg GO, GO-EV, or EV and the viability was measured at 24, 48, 72, and 96 h post-treatment. The data depicted represents the mean ± SD; ^†^*p* < 0. 05, ⋄*p* < 0.0005, relative to the control cells.

Visualization of EV-GO biomaterial was performed by scanning EM (SEM). SEM revealed a highly heterogeneous size composition of GO flakes (Figure 2). The larger sized GO flakes had lateral dimensions ranging from 20 to 40 microns, while the smallest sized flakes were less than 2 microns. The sGO flakes were much more homogeneous in size, with the majority of the flakes measuring less than 10 microns. Therefore, we selected sGO for further detailed assessments. On SEM, surface modifications were present on GO-EV and sGO-EV when compared with GO or sGO, which is consistent with successful conjugation of EV following the CuAAC reaction. The optical properties of GO- and sGO-based biomaterials were determined by absorbance spectrophotometry. A peak was detected at 257 nm at each of four different dilutions of GO, and absorbance intensity increased with increasing concentrations of GO (Figure 3).

### Effects of GO on Cell Viability

To evaluate the effects of GO on cell viability over time, HepG2 cells were treated with PBS or 0.4 μg GO for 24, 48, 72, or 96 h. A significant reduction in viable cell numbers was noted during incubation with GO compared with PBS at 24 hrs but not at subsequent time points. Notably, there was an increase in the number of viable cells with longer durations up to 96 h post-treatment. These observations suggest that HepG2 cells are able to overcome the cytotoxic effects of GO with more prolonged exposure.

### Cellular Effects of GO-EV and sGO-EV

**FIGURE 4:**
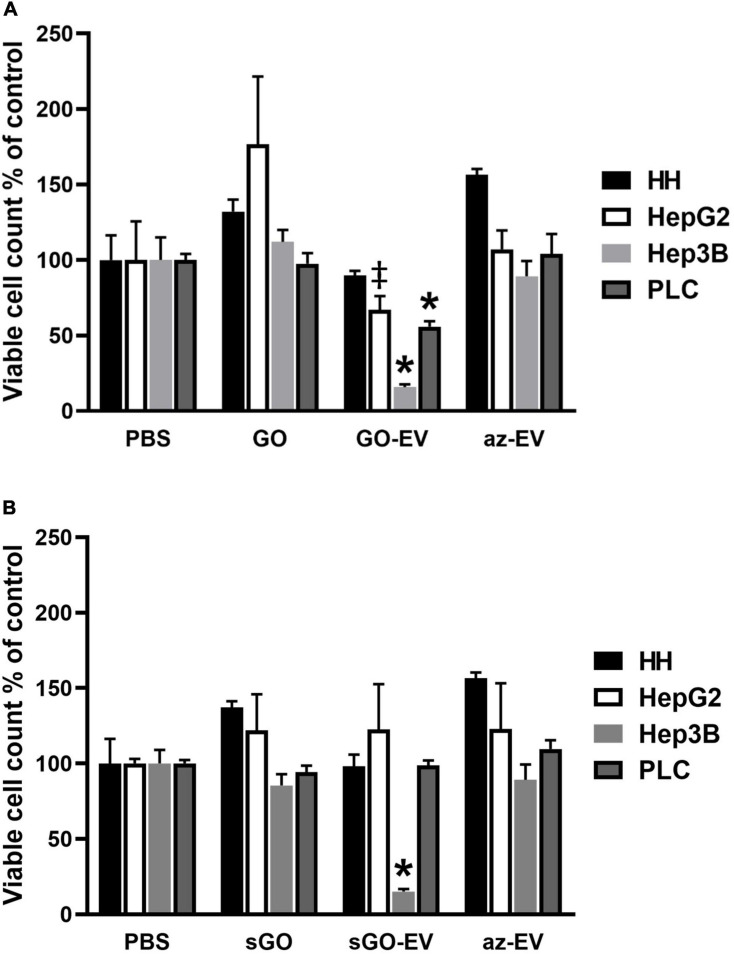
*C*ytotoxicity of GO-EV and sGO-EV. Healthy human hepatocytes (HH) as well as three malignant hepatocyte cell lines (HepG2, Hep3B and PLC) were incubated with PBS, 4 μg/ml GO, GO-EV, sGO, sGO-EV and az-EV. At 96 h post-treatment the viability of the cells treated with **(A)** GO- and **(B)** sGO-based biomaterials was evaluated by an MTS assay. The data represents the average ± SD. ^‡^*p* < 0.05, ^∗^*p* < 0.001 relative to PBS vehicle control.

To evaluate the effects of GO-EV in different cell types, cell viability was assessed in normal human hepatocytes (HH) and in HepG2, Hep3B, or PLC malignant hepatocyte cell lines after exposure to PBS, 4 μg/mL GO, GO-EV, or az-EV for 96 h. Although GO-EV did not reduce cell viability in HH cells, a reduction was observed in HepG2, Hep3B and PLC cells compared with PBS treated controls (*p* < 0.05, *p* < 0.001, *p* < 0.001, respectively) (Figure 4). Differences across cell lines were also noted, with Hep3B cells being the most sensitive to cytotoxic effects of GO-EV. These results suggest that cytotoxicity of GO-EV is cell type specific, with selective effects in malignant hepatocytes.

Synthetic approaches for GO can result in flakes of varying sizes. To evaluate whether these observed effects could reflect flake size, GO was first sonicated to prepare sonicated GO (sGO). Cell viability was assessed in HH, HepG2, and Hep3B cells treated with PBS, sGO, sGO-EV, and az-EV. Compared with controls, neither sGO nor sGO-EV altered cell viability in HH or HepG2 cells. However, sGO-EV, but not sGO treatment reduced viability in Hep3B cells after 96 h compared with PBS controls. These results suggest that exposure to GO-EV or sGO-EV can cause acute cytotoxicity in some HCC cell lines.

### GO-EV and sGO-EV Do Not Induce DNA Damage

**FIGURE 5:**
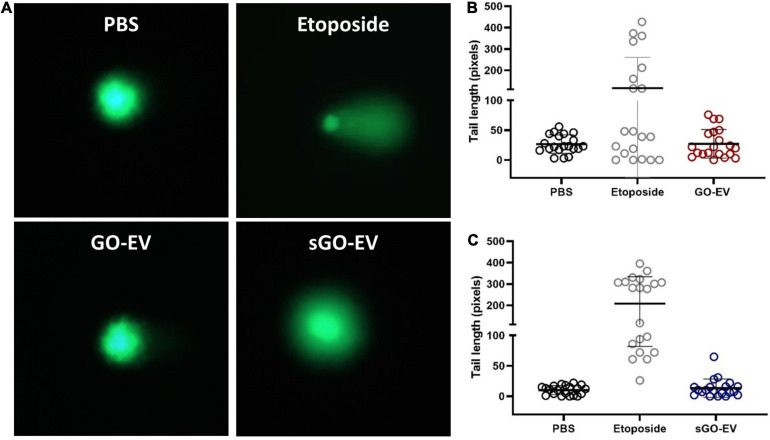
GO-EV does not induce DNA damage. HL-60 cells were treated with PBS (control), 4 μg/ml GO-EV or sGO-EV for 24 h, or 20 μm etoposide (positive control) for 1 h. An alkaline-based comet assay was performed at the treatment end point to assess for the presence of DNA breaks. Images of the DNA tails were captured by fluorescence microscopy **(A)** and the average tail length **(B,C)** was calculated for each treatment condition using the OpenComet software. The lines mark the average DNA tail length for each population.

Since GO-EV and sGO-EV reduced the viability of several liver cancer cell lines, we performed an alkaline-based comet assay to determine if the GO-based biomaterials could induce DNA strand breaks (Figure 5). Suspension cells are more sensitive to GO-induced toxicity, and thus we selected HL-60 cells to evaluate the genotoxic potential of GO-EV and sGO-EV treatment. There was no DNA damage observed in HL-60 cells treated with 4 μg/mL GO-EV (Figure 5). Similarly, treatment with 4 μg/mL sGO-EV did not induce any considerable genotoxicity. On the contrary, treatment with 20 μM etoposide, which served as the positive control, induced noticeable DNA damage.

### Biological Effects of GO-EV on Macrophages

**FIGURE 6:**
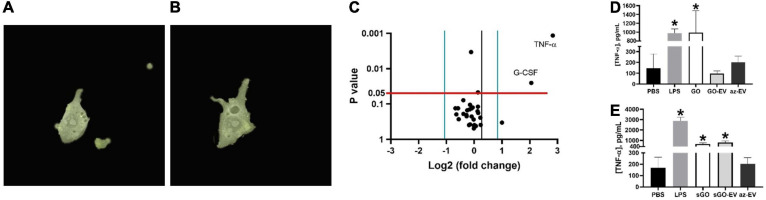
Effects of GO-EV on macrophages. **(A)** Time-lapse imaging of RAW264.7 murine macrophages incubated with PKH67 labeled GO-EV was performed. Images were captured **(A)** before uptake, and every 5 min following the addition of GO-EV to the cells. Imaging was ceased once the biomaterial was **(B)** successfully internalized by the RAW264.7 cells. Extracellular background masks were applied to the image. **(C)** RAW264.7 cells were incubated with PBS or 4 μg/ml GO-EV. After 3 h, conditioned media was collected, and assays were performed to determine protein concentrations for a panel of 31 chemokines or cytokines. Proteins with a log-two-fold or more change in concentration in response to GO-EV treatment were identified. A TNF-α ELISA was performed to quantify the TNF-α secretion by RAW264.7 cells treated with **(D)** GO- and **(E)** sGO-based biomaterials. RAW264.7 cells were treated with PBS, 10 ng LPS, 0.4 μg GO, sGO, GO-EV, sGO-EV, or az-EV for 24 h. Data represents average ± SD, ^∗^*p* < 0.001.

Macrophage phagocytosis was assessed by time lapse photography of biomaterial uptake by RAW264.7 murine macrophages incubated with 50 μL of PKH67 stained GO-EV or sGO-EV. First, we observed that RAW264.7 could take up GO-EV by phagocytosis (Figure 6). Next, we assessed whether GO-EV would affect cell viability or proliferation. Compared with PBS treated controls, treatment with 4 μg/mL GO-EV slightly enhanced RAW264.7 cell proliferation at 96 h (data not shown). Similar changes were observed in cells treated with 4 μg/mL GO or az-EV. To elucidate the effects of GO-EV treatment on RAW264.7 activity, chemokine and cytokine production was assessed following incubation with PBS or 4 μg/mL GO-EV for 3 h (Supplementary Table 2). In comparison to the PBS treated controls, there was a greater than two-fold increase in the secretion of tumor necrosis factor-α (TNF-α; *p* < 0.01) and granulocyte-colony stimulating factor (G-CSF; *p* < 0.05) in response to treatment with GO-EV. This suggests that exposure of RAW264.7 cells to GO-EV could induce the cells to differentiate into classically activated macrophages.

To confirm these findings and to evaluate the durability of TNF-α response, we performed a high sensitivity TNF-α assay in RAW264.7 cells incubated with PBS, 10 ng/mL lipopolysaccharide (LPS), 4 μg/mL GO, sGO, GO-EV, sGO-EV or az-EV for 24 h. In comparison to the PBS treated control cells, an increase in TNF-α secretion was observed in the RAW264.7 cells treated with GO, with levels similar to those observed with LPS (*p* < 0.001; Figure 6D). However, treatment with either GO-EV or az-EV alone did not increase TNF-α secretion. Similar to the effects observed in GO treated cells, sGO treatment also increased TNF-α secretion by RAW264.7 cells (*p* < 0.001). Similar increases in TNF-α secretion were also noted with sGO-EV (Figure 6E). These results indicated that the smaller-sized biomaterial elicit a more potent and longer-lasting immune response.

### Developmental Toxicity of GO-EV Treatment

**FIGURE 7:**
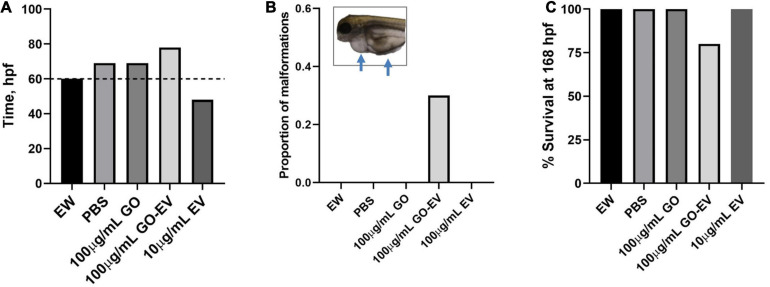
Developmental toxicity of GO-EV in zebrafish. At 24 h post-fertilization (hpf) zebrafish (*n* = 10) were incubated in embryo water (EW) alone or EW containing 100 μg/mL GO, 100 μg/mL GO-EV or 10 μg/mL EV every 24 h for a total of 168 h. **(A)** The time at which 70% of the zebrafish hatched following treatment with GO-based biomaterials was recorded from 45 to 85 hpf. **(B)** The zebrafish treated with GO-based biomaterials were monitored for the development of any malformations and the proportion of malformations present in each treatment group was calculated. Pericardial and/or yolk sac edema was observed in some zebrafish treated with GO-EV. **(C)** The survival of the zebrafish treated with GO-based biomaterials was recorded at 168 hpf.

To determine if GO and sGO-based biomaterials have *in vivo* effects, we evaluated their toxicity using zebrafish. Dechorionation and hatching in zebrafish takes place between 48 and 72 hpf. First, we monitored the hatching rate of the zebrafish starting at 45 hpf. In comparison to vehicle treated control zebrafish, there was a modest delay in the hatching rates of the zebrafish treated with 100 μg/mLGO and GO-EV, conversely, the rates in zebrafish treated with 10 μg/mL EV were accelerated (Figure 7). A similar acceleration in hatching was observed in the zebrafish treated with 10 μg/mL sGO, whereas the zebrafish treated with 10 μg/mL sGO-EV, 100 μg/mL sGO and 100 μg/mL sGO-EV all exhibited delayed hatching rates (Supplementary Figure 1). Next, we evaluated for the development of any malformations. In zebrafish exposed to GO-EV, yolk sac edema and pericardial edema were observed. Pericardial edema was also observed in the zebrafish treated with either low or high concentrations of sGO-EV. On the contrary, there were no malformations observed in the zebrafish treated with PBS, GO, or sGO at either 10 or 100 μg/mL. The heart rate varied considerably at different time points, but we did not observe any trends toward decreased heart rate in any treatment groups (data not shown). We further assessed survival of zebrafish at 168 hpf. A slight increase in mortality was observed in zebrafish treated with GO-EV, and with higher concentrations of sGO or sGO-EV but there was no lethality observed in zebrafish treated with PBS, GO or lower concentrations of sGO.

## Discussion

In the present study we have developed a graphene oxide-based biomaterial synthesized by copper-catalyzed cycloaddition of azide tagged bone marrow derived-MSC-EV to alkyne functionalized graphene oxide. This novel biomaterial offers the ability to combine the structural physicochemical benefits of graphene with the biological effects of MSC-EV. MSC-EV retain the intrinsic therapeutic properties of their parent cells and have shown to be effective in promoting tissue repair and regeneration, mitigating oxidative stress and modulating immune cell activities.

The utility of MSC-EV as acellular therapeutics is being increasingly recognized. Their use is enhanced by several properties. Their cargo can be altered by exogenous loading to selectively enrich them with modulatory agents such as anti-sense oligonucleotides (George et al., 2018), miRNAs (Pomatto et al., 2019) or siRNAs (Matsuda et al., 2019). In addition, selective manipulation of their content is feasible through genetic engineering of donor cells to express RNA or proteins of interest. Moreover, their surface can be engineered to express specific markers that facilitate tissue- or cell-targeted delivery of the EV. Furthermore, their cellular production can be modulated by microenvironmental perturbations (Yan et al., 2017; Zhang et al., 2020). Of particular therapeutic relevance, MSC-EV also retain an ability to home to sites of inflammation and injury, similar to their parental cells (Lai et al., 2013). For all of these reasons, MSC-EV are attractive acellular therapeutics as well as therapeutic delivery vehicles with the capacity for targeted delivery of bioactive therapeutic molecules.

The unique physicochemical properties and adaptability of graphene makes it attractive for development as a theranostic nanomaterial. Several biomedical applications such as drug and nucleic acid delivery, biosensing, photothermal, photodynamic therapy and tissue engineering have been proposed for graphene and its derivatives such as GO and rGO (Robinson et al., 2011; Hu et al., 2012; Lee et al., 2016; Shin et al., 2016; Vinothini et al., 2019). The oxygen-containing functional groups in GO and rGO contribute to their overall colloidal stability in aqueous solutions. These derivatives are often conjugated to polymers or other biomolecules in order to mitigate membrane-damaging effects or the effects of oxidative stress. Similarly, the conjugation of MSC-EV to GO may permit additional properties that can be exploited toward broader potential biomedical applications. The MSC-EV cargo contains a variety of bioactive molecules that can work alone, or in concert, to elicit a therapeutic effect (Liang et al., 2016; Yan et al., 2017). The modifiability of the EV cargo and the EV surface profile can contribute to achieve the desired biological effects in a targeted fashion (Ye et al., 2018). Furthermore, considering that EV and GO are internalized by different mechanisms, conjugation of EV to GO could enhance GO uptake by recipient cells (Huang et al., 2012; McKelvey et al., 2015). Thus, the biological effects of MSC-EV such as reducing tissue injury can be coupled with physical, biochemical or structural functionalities offered by graphene.

An advantage of conjugation of EV to GO allows exploitation of the properties of graphene, such as surface modifications for additional functional properties. For example, cytotoxic effects of GO-EV could be augmented by loading chemotherapeutic that are released in a pH-responsive manner in tumor settings (Ardeshirzadeh et al., 2015; Wang et al., 2019). Other potential applications may involve fashioning the GO-EV as a structural biomolecule for implantation as an extracellular scaffold within tissues such as bone or teeth, or within endoprostheses and stents placed in the body (Diomede et al., 2018; Li et al., 2018). In this context, the ability to selectively load MSC-EV exogenously after isolation, or endogenously through genetic or protein manipulation of the parental cells offer the potential ability to use GO-EV as a therapeutic delivery platform. For such applications, further studies to determine the kinetics of EV release from GO-EV would be valuable to determine whether controlled release of MSC-EV can be accomplished for therapeutic benefit.

The paucity of developmental or genotoxic effects of the GO-EV biomaterial paves the way for development of further applications in tissue engineering and regenerative medicine. Within the context of bone tissue engineering, GO elicits pro-osteoneogenic effects *in vitro* and *in vivo* (Hermenean et al., 2017). BM MSC-EV cargo has been shown to be enriched in several pro-osteogenic miRNAs (Ardeshirzadeh et al., 2015). Thus, GO-EV could augment the osteoinductive effects observed with GO. We observed variable effects on cytotoxicity of GO-EV and sGO-EV in liver cancer cell lines, though minimal cytotoxicity was observed in healthy hepatocytes. Notably, no genotoxicity was observed. Moreover, we did not detect any significant developmental toxicity in zebrafish. sGO-EV and GO-EV are readily recognized and phagocytosed by macrophages. Following their internalization by RAW264.7 cells, an alteration in the secretome profile with enhanced secretion of the pro-inflammatory cytokine, TNF-α was observed. The immunological impact of these biomaterials warrants further evaluation. Biocompatibility *in vivo* could be improved via the conjugation of GO with polymers that are capable of being cleaved upon internalization of the biomaterial to prevent the adverse accumulation of GO in cells (Li et al., 2014). Additional surface modifications may further reduce undesirable immune effects observed in our *in vitro* study.

This study developed a process for fabrication of a graphene-based biomaterial incorporating MSC-EV and examined their cytotoxicity and immunologic effects *in vitro* and developmental toxicity effects *in vivo*. GO-EV induced an inflammatory response and cell-specific cytotoxicity. While some developmental malformations were observed, these had a minimal impact on overall survival in zebrafish. There are opportunities to further improve the biocompatibility of GO-EV. For example, variable effects of cytotoxicity have been observed with GO in different study settings. Cytotoxicity can be influenced by the flake size and the degree of oxygenation of GO, with the smaller and more oxygenated forms of GO eliciting more potent cytotoxic effects (Pelin et al., 2017; Gurunathan et al., 2019). Differences in GO induced cytotoxicity have been observed between different malignant and non-malignant cells (Fiorillo et al., 2015). Attention to optimized approaches and selection of base materials is warranted in future studies because cytotoxic effects could be impacted by the physical differences in the lateral dimensions and overall shapes of graphene noted between top-down and bottom-up synthetic approaches (Lee et al., 2019). Such efforts are warranted to take full advantage of the use of GO-EV as a functional biomaterial that combines the versatility of graphene with the intrinsic therapeutic effects of cell derived EV for the development of biomedical applications.

## Data Availability Statement

The original contributions presented in the study are included in the article/Supplementary Material, further inquiries can be directed to the corresponding author/s.

## Ethics Statement

The animal study was reviewed and approved by the Mayo Clinic Institutional Animal Care and Use Committee.

## Author Contributions

TP contributed to the conception of the study and acquired the funding and resources. TP, JD, AM, and IY designed the methodology. JD, AM, and IY performed the experiments. JD and AM formally analyzed and curated the data. TP and JD wrote the original draft and revised and edited the manuscript. All authors contributed to the article and approved the submitted version.

## Conflict of Interest

The authors declare that the research was conducted in the absence of any commercial or financial relationships that could be construed as a potential conflict of interest.

## Abbreviations

az-EV: azide tagged extracellular vesicles
BM: bone marrow
CM: conditioned media
CuAAC: copper catalyzed alkyne-azide cycloaddition
EV: extracellular vesicles
GO: alkyne functionalized graphene oxide
hpf: hours post-fertilization
miRNA: micro RNAs
MSC: mesenchymal stem cells
rGO: reduced GO
sGO: sonicated GO
siRNA: small interfering RNA
TFF: tangential flow filtration
TNF: tumor necrosis factor.
