# Supplementary material for: Fabrication and Characterization of a Biomaterial Based on Extracellular-Vesicle Functionalized Graphene Oxide
Source: Front Bioeng Biotechnol. 2021 Jun 9;9:686510. doi: 10.3389/fbioe.2021.686510 (PMC8220207; doi:10.3389/fbioe.2021.686510)
Supplement: Supplementary file 1 [file Table_1.docx]

**Supplementary Information**

**Fabrication and characterization of a biomaterial based on extracellular-vesicle functionalized graphene oxide**

Julia Driscoll^1^, Anuradha Moirangthem^1^, Irene K Yan^1^, Tushar Patel^1*^

**
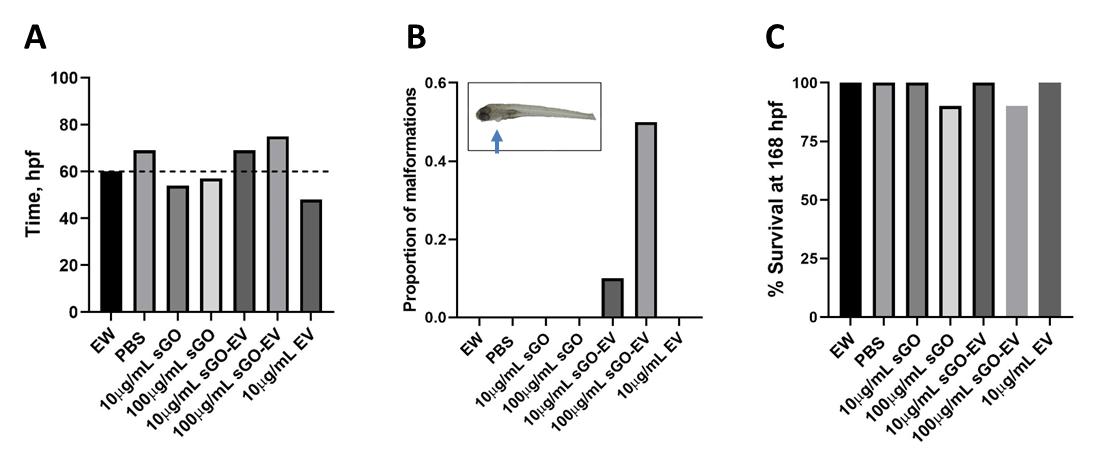
**

**Supplementary Figure 1: Developmental toxicity of sonicated GO (sGO)-based biomaterials in zebrafish.** At 24 hours post-fertilization (hpf) zebrafish (n=10) were treated with nothing (embryo water; EW), 1 or 10μg sonicated GO (sGO), 1 or 10μg sGO-EV or 1μg EV every 24 hours for a total of 168 hours. (**A**) The time at which 70% of the zebrafish hatched following treatment with sGO-based biomaterials was recorded from 45 hpf to 85 hpf. (**B**) The zebrafish treated with sGO-based biomaterials were monitored and the proportion of malformations observed were documented. Some zebrafish treated with sGO-EV developed heart edema. (**C**) The survival of the zebrafish treated with sGO-based biomaterials was recorded at 144hpf.

**Supplementary Table 1** Characterization of alkyne functionalized graphene oxide flakes

| **Number of layers** | **Flake size (µm)** | **Thickness (nM)** | **Purity (carbon to oxygen ratio)** |
| --- | --- | --- | --- |
| 1 | 0.5-5 | 1.1 | 75/35 |

**Supplementary Table 2** Changes in secreted proteins in cells incubated with GO-EV compared with PBS

| Cytokine/ chemokine | Fold change | p value |
| --- | --- | --- |
| Eotaxin | 0.964 | 0.125 |
| G-CSF | 4.159 | 0.025 |
| gm-csf | 0.642 | 0.196 |
| ifn- γ | 0.841 | 0.151 |
| IL-1α | 0.903 | 0.379 |
| IL-1β | 0.627 | 0.132 |
| IL-2 | 0.826 | 0.205 |
| IL-3 | 0.926 | 0.003 |
| il-4 | 1.006 | 0.395 |
| il-5 | 0.936 | 0.134 |
| il-6 | 0.896 | 0.143 |
| il-7 | 0.670 | 0.132 |
| il-9 | 0.993 | 0.492 |
| il-10 | 0.829 | 0.222 |
| il-12 (p40) | 0.765 | 0.191 |
| il-12 (p70) | 1.044 | 0.421 |
| il-13 | 1.000 | N/A |
| il-15 | 0.680 | 0.270 |
| il-17 | 0.994 | 0.408 |
| ip-10 | 1.106 | 0.179 |
| kc | 0.880 | 0.395 |
| lif | 1.098 | 0.047 |
| lix | 2.000 | 0.335 |
| mcp-1 | 1.178 | 0.391 |
| m-csf | 0.902 | 0.105 |
| mig | 1.166 | 0.245 |
| mip-1α | 1.099 | 0.119 |
| mip-1 β | 1.090 | 0.161 |
| mip-2 | 0.763 | 0.078 |
| rantes | 0.99 | 0.225 |
| tnf-α | 7.126 | 0.001 |
| vegf | 0.989 | 0.275 |

Abbreviations: G-CSF, granulocyte colony stimulating factor; GM-CSF, granulocyte-macrophage colony stimulating factor; IFN, interferon; IL, interleukin; KC, keratinocytes derived chemokine; LIF, leukemia inhibitory factor; LIX, C-X-C motif chemokine 5; M-CSF macrophage colony stimulating factor; MCP, monocyte chemoattractant protein-1; MIG, monokine induced gamma interferon; MIP, macrophage inflammatory protein; TNF, tumor necrosis factor; VEGF, vascular endothelial growth factor
